# Implementation of primary palliative care interventions across six sub-Saharan African countries: understanding contextual determinants and lessons learnt

**DOI:** 10.1136/fmch-2025-003490

**Published:** 2026-09-10

**Authors:** Anna Peeler, Oladayo Afolabi, Kennedy Nkhoma, Catherine Evans, Eve Namisango, Mary Abboah-Offei, Lindsay Farrant, Joy Hunter, Edwina Beryl Opare-Lokko, Adwoa Boamah Mensah, Maya Jane Bates, Dorothee van Breevoort, Duncan Kwaitana, Modai Mnenula, Dickson Chifamba, Lovemore Mupaza, Richard Harding

**Affiliations:** 1 Cicely Saunders Institute of Palliative Care, Policy and Rehabilitation, King’s College London Florence Nightingale Faculty of Nursing Midwifery & Palliative Care, London, England, UK; 2 African Palliative Care Association, Kampala, Central Region, Uganda; 3 School of Health and Social Care, Edinburgh Napier University, Edinburgh, Scotland, UK; 4 University of Cape Town, Rondebosch, WC, South Africa; 5 School of Nursing and Midwifery, Kwame Nkrumah University of Science and Technology, Kumasi, Ghana; 6 Department of Family Medicine, Kamuzu University of Health Sciences, Blantyre, Southern Region, Malawi; 7 Island Hospice and Healthcare, Harare, Zimbabwe

**Keywords:** Primary Health Care, Palliative Medicine, Health Resources

## Abstract

**Objective:**

To synthesise five purposively selected service-level primary palliative care interventions in six sub-Saharan African countries to identify the contextual barriers and facilitators and the implementation strategies employed to address them.

**Setting:**

Primary palliative care studies that have taken place in sub-Saharan Africa in the last 5 years.

**Design:**

We used an implementation-focused formative evaluation design involving semi-structured interviews with every site lead (n=7) supplemented by a review of study-related documentation. Data were analysed using framework analysis underpinned by the relevant constructs of the Consolidated Framework for Implementation Research.

**Participants:**

Site leads from each of the five studies were interviewed.

**Results:**

Facilitators to implementing primary palliative care interventions included the following: (1) the relative advantage compared with standard care (ie, person-centred approach, expansion to those with non-cancer conditions), (2) primary care worker champions within sites, (3) broad sustained engagement with policymakers, managers, healthcare workers, patients and families and (4) availability of linked healthcare records. Barriers included the following: (1) inconsistent staffing and training needs for the diverse workforce, (2) fragmented referral pathways, (3) inconsistent national and local policies and (4) physical infrastructural limitations.

**Conclusion:**

Context-specific facilitators, barriers and recommendations should be considered to bolster the implementation process of future primary palliative care interventions. Advocacy is needed to ensure adequate investments in the design, equipping and staffing of primary healthcare in sub-Saharan Africa to properly support patients and their families.

**WHAT IS ALREADY KNOWN ABOUT THIS TOPIC**

- Delivering palliative care within primary healthcare offers a potential effective and feasible solution to meet the growing palliative needs in Africa.
- Context constitutes a major layer of complexity in the implementation of complex interventions such as delivering palliative care within primary healthcare.
- Interventions aimed at improving primary palliative care delivery should reflect the unique cultural and socioeconomic context of the communities in which they are implemented.

**WHAT THIS STUDY ADDS**

**HOW MIGHT THIS STUDY AFFECT RESEARCH, PRACTICE OR POLICY**

- This cross-country synthesis of facilitators and barriers offers a practical guide for researchers and implementers to anticipate and respond to common challenges when scaling palliative care in similar contexts across sub-Saharan Africa and other LMICs.
- This study will inform health system strengthening efforts and guide workforce development initiatives to encourage incorporation of mentorship for healthcare workers, early and sustained stakeholder engagement and incentives for interventionists.
- Findings highlight the need for coherent, well-communicated national policies and sustainable funding models to support primary palliative care. Policymakers can use this evidence to align palliative care strategies with broader primary healthcare goals and Universal Health Coverage agendas.

**WHAT IS THIS RESEARCH FOCUSED ON EXPLORING, VALIDATING OR SOLVING?**

**WHAT CONCLUSIONS DID THIS RESEARCH DRAW THROUGH DESIGN, METHOD AND ANALYSIS?**

- Through a cross-country synthesis of implementation experiences, the research identified common and context-specific factors that facilitate or hinder the integration of palliative care within primary healthcare. Findings demonstrate that implementation cannot rely on a one-size-fits-all approach but several recurring strategies can strengthen implementation across settings. These include mentorship and ongoing support for healthcare workers, early and sustained stakeholder engagement, appropriate incentives for those delivering interventions, coherent and well-communicated national policies and sustainable funding mechanisms. The analysis provides pragmatic recommendations for adapting and refining primary palliative care interventions in sub-Saharan Africa and potentially other low-income and middle-income countries.

**WHAT IS THE VALUE, MEANING AND IMPACT OF THIS RESEARCH? IS THERE ANY FOLLOW-UP STUDY BASED ON THIS RESEARCH?**

- The research provides a practical evidence base for researchers, implementers and policymakers seeking to integrate and scale palliative care through primary healthcare systems. By identifying barriers that can be anticipated and facilitators that can be deliberately incorporated into implementation strategies, the findings can inform intervention design, health-system strengthening, workforce development and national palliative care policy. In particular, the research highlights opportunities to align palliative care implementation with broader primary healthcare and Universal Health Coverage agendas, supporting more sustainable approaches to expanding access in resource-constrained settings.
- The findings also provide a foundation for future implementation and scale-up studies that test how these recommendations can be operationalised across different health-system contexts, including evaluating strategies for workforce mentorship, stakeholder engagement, financing and policy implementation.

## Introduction

 Serious health-related suffering, or suffering associated with illness or injury that substantially impairs a person’s physical, psychological, social or spiritual well-being, is expected to increase substantially over the next few decades as populations live longer.^1^ The rise will be greatest in low- and middle-income countries (LMICs), where numbers will more than double by 2060.^2^ In these countries, palliative care, or the active holistic care of individuals across all ages with serious health-related suffering, is often unavailable, inadequate or inaccessible.^3 4^

The WHO has consistently emphasised that palliative care is an essential component of Universal Health Coverage and should be integrated across all levels of the health system, including primary care.^5 6^ By embedding palliative care within primary care settings, countries can expand access to holistic, person-centred care for people living with serious health-related suffering. This is particularly important in settings where specialist palliative care services are limited.^7^ This approach aligns with WHO calls for comprehensive palliative care that addresses physical, psychological, social and spiritual needs throughout the illness trajectory.^8^

In LMICs and particularly in sub-Saharan Africa, primary care represents the most accessible and equitable platform for delivering palliative care. However, translating global policy recommendations into practice remains challenging due to contextual constraints such as workforce capacity, limited infrastructure, sociocultural norms, inadequate financing and competing health system priorities. Although a small but growing body of literature demonstrates that primary palliative care is feasible and acceptable in sub-Saharan African settings,^9 10^ there is limited cross-country evidence examining how contextual factors shape implementation success.^11 12^

This study is therefore relevant in addressing a critical implementation gap. By synthesising lessons from five primary palliative care intervention studies conducted across six sub-Saharan African countries, we aim to identify common contextual barriers and facilitators and the implementation strategies used to address them. This will provide a consolidated, practical guide for researchers and policymakers aiming to scale primary palliative care in similar low-resource settings. In doing so, this work contributes practical, contextually grounded evidence to support the translation of WHO’s vision for comprehensive palliative care into scalable primary healthcare practice in sub-Saharan Africa and other low-resource settings.

## Methods

### Study design

This project began as a 5 year collaboration starting in June 2018 between researchers at King’s College London, the African Palliative Care Association and universities in Ghana, Malawi and Zimbabwe aimed at expanding access to primary palliative care in sub-Saharan Africa. The studies included in this synthesis (seen in table 1) were purposively selected from within this collaboration to enable an implementation-focused formative evaluation, as detailed implementation documentation and direct access to implementation leads were required to support in-depth analysis using the Consolidated Framework for Implementation Research (CFIR) 2009 version.^13^ The intent was not to identify all primary palliative care interventions in the region but to synthesise implementation lessons from a defined set of studies for which rich contextual data were available.

**Table 1:** Summary of included studies

Title	Country sites	Timeline	Language(s)	Illness focus	Study design	Theoretical underpinnings
Integrated Primary Palliative Care Intervention in Nigeria^5^	Nigeria	2019–2022	English, Yoruba	Any serious illness	Exploratory mixed-methods design in three stages: Qualitative descriptive study Intervention development workshop using theory of change Intervention training feasibility using pretest/post-test with embedded focus group discussion	MRC framework for the testing and evaluation of complex interventions^34^ Theory of change^17^ Kirkpatrick’s Model for Evaluating Educational Outcomes^35^ Integrative model of patient-centredness^36^
HeAlth Systems StrEngThening in sub-Saharan Africa (ASSET) Integrated palliative care and primary health care^37^	South Africa	2019–2022	English, Afrikaans, Xhosa	COPD	Cluster stepped wedge hybrid type two feasibility RCT	MRC framework for the testing and evaluation of complex interventions^34^ MRC guidance for process evaluation of complex interventions^38^ Theory of change^17^
Multimorbid Ageing Primary Palliative Care (MAPCare)^39^	Ghana, Malawi, Zimbabwe	2021–2023	English, Chichewa, Shona, Twi	Multimorbidity	Sequential mixed-methods design in two phases: Qualitative interviews and theory change workshop using codesign Feasibility testing using prospective cohort design; programme evaluation and non-participant observational assessment	MRC guidelines for development and testing of complex interventions^34^ MRC guidance for process evaluation of complex interventions^38^ Theory of change^17^
Person-centred care for people living with HIV^18^	Ghana	2018–2019	English, Twi	HIV/AIDS	Sequential mixed-methods design in three stages: Qualitative descriptive study Cluster randomised feasibility trial design with embedded interviews for process evaluation	MRC framework for the testing and evaluation of complex interventions^34^ Mezzich’s theory of person-centred care^40^ Theory of change^17^
Health Systems Strengthening through Person-Centred Care	Uganda, Zimbabwe	2020–2021	English, Luganda, Runyakitara, Luo	Any serious illness	Sequential mixed-methods design in three stages: Qualitative in-depth interviews Expert opinion workshop using theory of change Cognitive interviews and cross-sectional study for development and validation of person-centred care measure	Person-centred care in practice^41^ WHO conceptual model of palliative care development^42^ Theory of change^17^

COPD, chronic obstructive pulmonary disease; MRC, Medical Research Council; RCT, randomised controlled trial.

We used an implementation-focused formative evaluation design to describe and understand major contextual factors, barriers and facilitators to implementing primary palliative care in sub-Saharan Africa.^14^ This involved a two-stage process of semi-structured interviews with site leads underpinned by the domains and relevant constructs of the CFIR (seen in table 2) and a review of study-related documentation from each site.^13^ Each included study received ethical approval from the institutional review board at King’s College London and the country and institution-specific regulatory bodies at each of the study sites. Additionally, this synthesis project received ethics approval from King’s College London (MRA-22/23-35071), and site leads were asked to give informed consent to participate in interviews.

**Table 2:** CFIR domains and related constructs

Domain	Description	Constructs
Intervention characteristics	The innovation or service that is being implemented, how it was developed and by whom, its complexity and adaptability and its cost.	Source of the intervention, evidence-base, relative advantage, adaptability, trialability, complexity, design and cost
Individual characteristics	The roles and involvement of leaders and facilitators at all levels throughout the development and implementation of the intervention	High-level leaders, mid-level leaders, opinion leaders, implementation facilitators, site leads, implementation team members, other implementation support, innovation deliverers, innovation recipients, need capability, opportunity and motivation
Outer setting	The larger context in which the intervention is being delivered, including critical incidents caused by outside influences, local attitudes, conditions and policies that influence the intervention implementation and external pressure that guide its delivery	Critical incidents, local attitudes, local conditions, partnerships and connections, policies and laws, financing, societal pressure, market pressure and performance-measurement pressure
Inner setting	The local setting in which the intervention is implemented, including physical and technological infrastructure, staff workflow, relationships and values and the tension for change	Physical infrastructure, information technology infrastructure, work infrastructure, relational connections, communication, human equality-centredness, recipient-centredness, deliverer-centredness, learning-centredness, tension for change, compatibility, relative priority, incentive systems, mission alignment, available resources (ie, funding, space, materials and equipment) and access to knowledge and information
Implementation process	The strategies employed to implement the intervention, including building successful implementation teams, needs assessment and continual evaluation and adaptation as needed	Teaming, assessing needs and engaging innovation deliverers, assessing needs and engaging innovation recipients, planning, tailoring strategies, doing, reflecting and evaluating implementation and adapting

CFIR, Consolidated Framework for Implementation Research.

### Sample and data collection

At least one leader from each site was purposively sampled, meaning that some studies had multiple site leads interviewed. OA and AP conducted semistructured interviews using an interview guide underpinned by the relevant constructs of the CFIR framework. Both OA and AP are nurse researchers with extensive qualitative research methods training and have experience conducting and analysing qualitative interviews. In addition to the questions about the CFIR domains, the semistructured interview guide also included additional questions on implementation lessons learnt from the projects. Interviews were conducted remotely on Microsoft Teams, in English, and were not recorded but involved detailed note taking by OA and AP to capture the perspective of the respondents. The decision to not audio record the interviews was made deliberately to support rapport building, encourage candid reflection and align with the analytic aims of the study. On average, interviews lasted about 65 min. To enhance rigour, respondents were sent interview notes for member checking and were given the opportunity to correct or provide additional comments. Even so, member checking yielded only very small changes or clarifications to the notes (ie, spelling errors, adding additional context). Non-participant-based study documentation such as summary reports were also reviewed for relevant information to capture different domains of the CFIR framework.

This study applied the concept of data sufficiency to determine the adequacy of data collection.^15^ Data collection was deemed sufficient when all involved site leads had been interviewed, all CFIR domains and relevant constructs were adequately represented across sites and no additional implementation-relevant insights were identified that altered interpretation of the findings.

### Data analysis

Framework analysis was used to analyse collected data.^16^ A template including all the constructs of the CFIR was created as an a priori analytical framework. After reading the interview notes and study documents to become familiar with the data, two analysts (OA and AP) independently coded each item. Coding followed a deductive approach guided by the definitions of the constructs in the CFIR framework. Discrepancies in coding were resolved through consensus discussions between the two analysts, with a third researcher available for arbitration if needed. To facilitate cross-system comparisons, we examined contextual determinants that acted as barriers or facilitators to the implementation. To do this, we explored common themes, key differences and how each relates to the unique cultural and socioeconomic context in which the interventions were implemented. Based on the successes and challenges each study team faced when implementing their intervention, we developed a list of recommendations to guide researchers and clinicians aiming to improve delivery of primary palliative care services in sub-Saharan Africa. Study documents were used to provide context or detail that triangulated data captured in the interviews with site leads.

## Results

The earliest of the five included studies began in 2018, and all finished by March 2023 or before. Overall, 593 patients, families and primary healthcare workers participated in the included studies from six African nations (two studies were conducted in multiple countries), speaking 11 different languages. Four of the five studies incorporated qualitative interviews to inform the intervention development, evaluation and implementation. One study (ASSET in South Africa) adapted an intervention that had been previously developed. Study documents analysed included published results (n=4), PhD dissertations (n=2), research-in-progress presentations (n=2), unpublished protocols (n=4), published protocols (n=2), intervention standard operating procedures (n=3), grant applications (n=2) and news articles and other grey literature (n=3).

The five included studies were conducted in eight sites across six countries (seen in table 1). As one site lead managed the two sites for the two studies conducted in Zimbabwe, seven site leads were interviewed across the included studies. Interviews lasted between half an hour and 2 hours. The participants included two men and five women, in roles including nurse researchers (n=3), epidemiologist (n=1) and a physician (n=3). One person was a site lead for multiple studies in their country.

**Figure 1:**
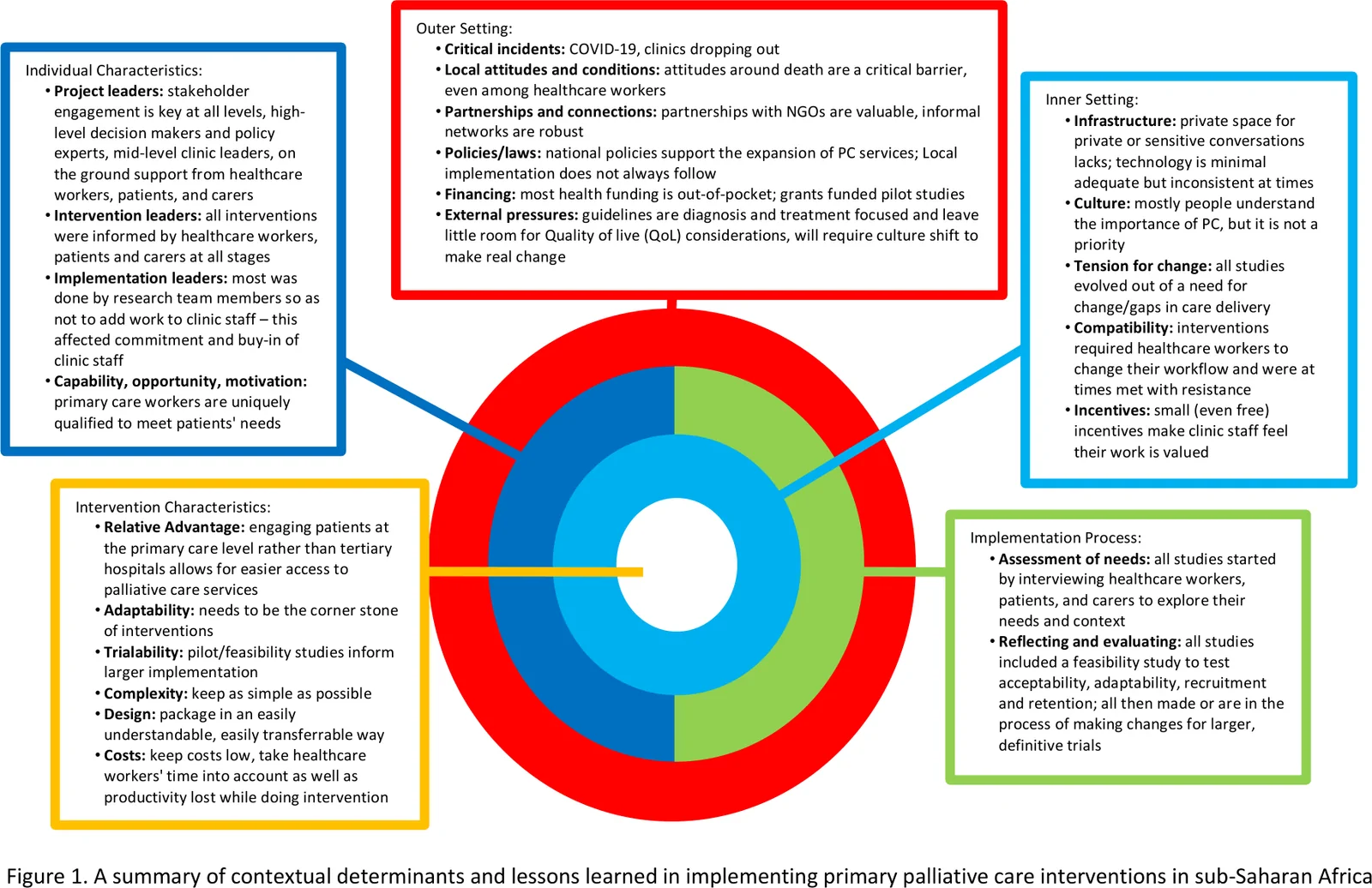
A summary of contextual determinants and lessons learnt in implementing primary palliative care interventions in sub-Saharan Africa. NGOs, non-governmental organisations; PC, palliative care.

Below, we present a summary of the contextual determinants (seen in figure 1) encountered by the five study teams followed by a detailed synthesis of each domain of the CFIR framework, as well as successes and challenges the study teams experienced and recommendations for implementing primary palliative care interventions in sub-Saharan Africa (table 3).

**Table 3:** Successes and challenges in implementing primary palliative care interventions

Successes	Challenges	Recommendations
Holistic interventions improve outcomes for both patients and carers, such as QoL, carer burden, knowledge of palliative care Pilot studies suggest that interventions are feasible and acceptable (ie, are able to meet recruitment targets, train staff and embed in existing workflows) Patients and carers feel most comfortable around people they already know and who work in their communities, like primary healthcare workers or community nurses Creating networks requires a lot of initial planning and work but pays off exponentially throughout the project Mentorship for primary healthcare workers participating in interventions was key in obtaining and maintaining clinic buy-in Incentives (ie, cash payments, certificates of completion, access to university resources) made clinic/intervention staff feel appreciated	Resource limitations (ie, staffing and supply shortages, space limitations and donor dependence) Environments that discourage discussing death and dying, even in a healthcare setting Establishing and maintaining relationships with recruiting clinics and healthcare staff requires significant effort at the start; this is made more challenging if research team members are not in-country Mid-level buy-in from clinic staff can be especially difficult, particularly if the intervention changes workflows or adds additional tasks Sustainability (ie, securing time, effort, resources and financing) Patients often defer to providers’ recommendations; the power imbalance between healthcare workers and patients is entrenched and limits shared decision making Staffing, regulatory constraints and increased strain on health systems during the COVID-19 pandemic Political instability and a lack of political engagement slows bureaucratic approvals and compromises funding	Include sensitisation exercises that educate patients and families about the purpose and benefits of palliative care; reinforce personal advocacy and patient-centred care Establish networks early in the intervention development phase; plan for sites to drop out Engage high-level leaders in order to secure sustainable funding and impact local and national government policies Provide incentives for patients, families and healthcare workers involved in the intervention Build additional time into timelines to allow for unexpected delays Make physical space for consults and sensitive conversations

QoL, quality of life.

### Intervention characteristics

The included interventions were all complex interventions designed with strong theoretical underpinnings (seen in table 1), informed by patients, family members and healthcare workers with relevant lived experiences, and evaluated by evidence-based outcomes of interest. A summary of intervention components can be seen in table 4. The relative advantage of each intervention typically stemmed from the limited access and poor quality of palliative care provision prior to the intervention being implemented. Each intervention emphasised the person-centred approach and broadened the population who should have access to palliative care services (eg, groups historically underserved by palliative care in these contexts, such as those with chronic obstructive pulmonary disease, heart failure, HIV/AIDS and/or multimorbidity). One of the site leads summarised this well, saying,

Older patients with multiple chronic conditions are often overlooked for the palliative care needs. Often, chronic conditions are treated in silos without regard for the complicated interactions between diseases, medications, and treatment plans. (Site Lead 5)

All the interventions were designed to accommodate the complex needs of patients living with a life-limiting illness and fit within the local context, though with somewhat varying methods and hypothesised mechanisms of action. Even so, each intervention followed a similar pattern of needs assessment, education and mentorship.

The major elements included healthcare provider education and mentorship, comprehensive needs assessments for patients and families and an emphasis on patients’ and family caregivers’ support and education. (Site Lead 1)

For each study, this required coordination between different levels of care (tertiary, secondary and primary) through strengthening existing referral linkages or designing new linkages. While all the studies involved the development of theory of change,^17^ adaptability and flexibility to ensure the interventions can respond to unique patient needs and priorities was a common theme through all the studies.

Ensuring limited cost to patients was key to the successful uptake of the interventions. While some costs are limited to the beginning of the study (ie, intervention set up, healthcare worker training), others are ongoing (ie, cost of care and supplies). Most of the studies had limited or no healthcare insurance or cost-protected access to healthcare. Using existing resources meant that the costs for the interventions varied between sites depending on the interventions’ components and were limited to the clinicians’ time to take training and deliver the care. One site lead commented on the various costs associated with palliative care interventions, saying:

There are many costs involved that would need to be funded externally—providers' time is expensive, and clinics often can't afford to pay them for time spent training; reducing workload so that care providers have more time to listen to patients; provision of appropriate structures (ie, consultation rooms, patient rooms, removing of stairs to help patients with COPDs move with ease, to facilitate person-centred care). (Site Lead 6)

Nevertheless, site leads highlighted the importance of the intervention being embedded in the healthcare service plan in a way that minimised burden on the healthcare workers and disrupted existing services to ensure sustainability.

### Individual characteristics

All the sites acknowledged the importance of stakeholder engagement at all levels, including high-level decision makers, policy experts and mid-level clinic leaders. This buy-in goes beyond getting administrative approvals but requires ensuring their understanding of the concept of palliative care and the need for it within primary healthcare. One participant highlighted the role of early stakeholder engagement, saying:

An opinion leader who championed palliative care development in the country was also interested in the implementation and supported access to gatekeepers at different levels including the state ministry of health and local governments. (Site Lead 1)

The ease of finding collaborators and obtaining buy-in varied across sites. Some sites engaged local opinion leaders (ie, community and religious leaders) with influence and interest in palliative care as partners in the project to ensure access to gatekeepers and validate the need for the intervention implementation, while one site did not. Beyond opinion leaders who might sway public opinion, multiple site leads discussed the importance of engaging clinic-level primary healthcare workers early to gain an understanding of palliative care, its potential benefits for patients and its potential benefits for the larger healthcare system. They found that primary healthcare workers who understood holistic care were more motivated to fully participate in the interventions. Even so, they also acknowledged that despite feeling motivated to incorporate palliative care, time and resources can limit delivery. One said,

When [healthcare workers] understand the importance of holistic care, they usually take the time required to incorporate it into their plan of care. Time and resource limitations can cause them to revert back to the old workflow without sustainability measures in place. (Site Lead 7)

Conversely, the impact of not engaging primary healthcare workers was clear in one study where a clinical site dropped out prior to the start of the intervention. This Site Lead hypothesised that this was likely because they were unable to sustain enthusiasm for the intervention through the COVID-19 pandemic without committed supporters within the clinic site.

To achieve this, multiple studies incorporated training and mentorship for healthcare workers such as doctors, nurses, clinical officers and social workers to facilitate implementation while a member of the research team provided support where needed. This was done to improve sustainability by further embedding the intervention into the clinic workflows to ensure the healthcare workers felt ownership of the implementation. One site used a mixture of research team members and trained healthcare workers on the frontline to facilitate the intervention.

According to all site leads, primary healthcare workers are the perfect candidates to meet the palliative care needs of patients as they are closest to patients in the community. Primary healthcare workers already receive general symptom assessment and management training, so they only require some additional training in the fundamentals of palliative care, mentorship by experienced providers and workplace incentives to ensure their engagement with the palliative care interventions. One site lead said,

Primary healthcare providers are uniquely positioned to deliver this intervention. They are community-based, know their patients well, and are respected in the community. They just need a little extra training and incentive to conduct holistic assessment and work the patient’s needs into the plan of care. (Site Lead 3)

Even so, context-specific workforce limitations and scope of practice laws were mentioned as crucial to consider in the development of interventions. Clinicians were inconsistently available across the sites, and facilities were staffed by different cadres of healthcare workers with varying levels of qualification, scope of practice and professional accountability. For example, community healthcare workers in some countries could develop care plans within established guidelines when there are no physicians available, while in others they could only provide social support and help with basic nursing tasks.

### Outer setting

Many external factors influenced the implementation of each intervention. The COVID-19 pandemic had a major impact on the implementation of all but one of the studies. Site leads detailed how the pandemic created an increased demand for palliative care services, coupled with staff shortages and burnout that made it difficult to deliver high-quality person-centred care. Only one study^18^ was not impacted as it was completed prior to the start of the pandemic. Leads stated that they were forced to accept the global halt in clinical research and provided limited advice for how to navigate similar incidents should they occur in the future. Beyond the COVID-19 pandemic, one participant discussed how political instability exacerbated funding uncertainties, restricted people’s movement and limited their ability to access healthcare services.

Socio-cultural issues such as hesitancy to discuss death and dying, stigma associated with living with a life-limiting condition and misconceptions about palliative care were preponderant across the sites. Furthermore, the gendered stereotype of caring and the lack of caregiving support from men was also identified as a barrier. One site lead listed barriers to delivering care resulting from local attitudes, saying:

Negative attitude towards death and dying. The caring role is biased towards women, men may not support caregiving even when they can*.* (Site Lead 6)

Next, site leads discussed the impact of national and local policies on their work. Multiple site leads considered how healthcare policies and metrics in host countries are often more focused on diagnosis and curative treatment as opposed to multidimensional, holistic care that prioritises quality of life concerns. This created barriers as study sites were sometimes forced to prioritise curative models of care over the palliative models emphasised in the interventions. One site lead lamented the fact that even where national benchmarks exist to measure palliative care delivery performance, they were not always prioritised in routine care, saying:

[country] has national policies related to palliative care access and delivery—these are not always implemented in practice. This intervention was designed in part to help meet these national benchmarks. (Site Lead 2)

Furthermore, national policies supporting the availability of opioids and other essential palliative care medications were a major facilitator to implementing the interventions. For example, one country’s national drug control policy advocates for wide availability and accessibility of opioids for palliative care. This eased implementation as research teams did not have to navigate a complicated landscape of drug procurement and delivery. The site lead detailed:

the political environment is favourable… We have standards for palliative care, we also have an accreditation system for facilities that are allowed to stock morphine in the pharmacy, trained nurses can prescribe morphine, morphine is reconstituted locally. (Site Lead 6)

Every lead discussed how despite national palliative care or disease-specific policies that recommended palliative care integration across all levels, there is often a lack of guidance about which patients should be prioritised and lack of resource pledging to ensure local services reach the patients who need them. Earmarked government funding to palliative care services is essential for sustainability of interventions. Multiple site leads went on to highlight that research is often easier to fund than sustainable healthcare services as they are typically time-bound, focused on one area or disease and supported by international funders. In situations where government funding was insufficient, site leads detailed how they developed strong connections with local non-governmental and for-profit organisations who donate time, personnel, resources and supplies to support patient care. This highlighted the need to formalise networks into established and consistent partnerships to which patients can be referred for the meeting of social needs in order to create sustainable services.

Finally, referral pathways were discussed by every site lead as both a facilitator and a barrier to implementing complex primary palliative care interventions. Across sites, referral linkages and networks with external organisations were not always available or robust. Some sites had defined referral pathways between primary, intermediary and specialist palliative care providers, standalone palliative care training institutions associated with clinical sites, referral teaching hospitals linked with academic institutions and specialised palliative care units for patients with complex needs. This meant that patients with known palliative care needs were able to be referred to a higher level of care. However, the referral pathways from tertiary healthcare facilities back to the primary healthcare were not always well defined and were non-existent in some settings. This meant that patients who were seen or diagnosed by a specialist often did not know where they could follow-up or receive continued primary care.

### Inner setting

Study sites encountered substantial obstacles due to physical infrastructure issues and healthcare workers’ workflow. Lack of private spaces, technological limitations and ineffective accessibility considerations in facility design were discussed by all site leads as barriers to delivering the interventions. This limits ill patients’ ability to enter facilities, especially those with frailty or physical disability, in order to access care and healthcare workers’ ability to have private sensitive conversations with patients and their families.

Furthermore, various workflow-related factors made implementation challenging at some sites. Staffing shortages meant that healthcare workers found it difficult or were unable to take time outside of their work schedules to participate in training for the interventions. One participant recalled,

Work infrastructure did NOT always support the intervention—accepted workflows, healthcare provider preferences, staffing etc made it difficult to provide the training and make substantial culture change within the clinics. Specifically, one clinic was understaffed and could not allow their staff to attend two hours of training for the intervention, so instead they attended the training in an abbreviated format over one hour. (Site Lead 2)

Site leads attempted to combat this by setting reasonable expectations for those who would deliver the intervention, by building in time and financial incentives for training and by making the interventions as unintrusive as possible without sacrificing quality. Overall, fitting interventions into existing workflows was an important consideration and required sustained commitment and continual refinement throughout the studies.

Outside of the time and energy required for training, the resources required to deliver the intervention (ie, additional time for comprehensive assessments, additional supplies) had the potential to disrupt the standard workflow in clinic sites. The need for comprehensive assessment and the nature of conversation with patients with serious illness were perceived as increasing pressure on healthcare workers’ time. At sites which relied on health insurance reimbursement, healthcare workers raised concerns that the slower pace of consultations would decrease revenue. In addition, interventions often required a substantial mental and cultural shift in their way of working, creating additional emotional load.

Across sites, information technology infrastructure also created barriers to implementing interventions. In all but one site, information technology infrastructure (ie, electronic medical records, data sharing applications and digital registers for data aggregation) was inadequate for appropriate record keeping. Furthermore, variations in medical record keeping between sites made data capture and sharing challenging. Multiple site leads asserted that having a consistent electronic medical record system would have facilitated easier and better data collection, storage and access for continuity and consistency of care.

Finally, the perception of the alignment of palliative care to the mission of primary healthcare and the relative priority they placed on delivering the intervention varied among sites. At times, the primary healthcare workers (ie, nurses, community health workers (CHWs), physicians and other primary care clinic staff) delivering the intervention did not feel that palliative care for patients with serious illnesses aligned with their values. In the face of competing priorities, implementation of primary palliative care intervention suffered or was forgotten. One participant stated,

Relative priority varied based on the provider—some people were really hesitant, while others were very enthusiastic about it. When the clinic leaders feel the intervention is really important, they can sell it to the rest of the clinic employees. (Site Lead 3)

As such, multiple site leads asserted that they overcame this mismatch of priorities by recruiting managers who were passionate about primary palliative care and providing extensive education about the purpose and benefits for those who were not. They found that this mid-level buy-in was essential to convincing the rest of the clinic’s employees that the intervention was worth delivering consistently and sustaining implementation. Furthermore, incentives for stakeholders, managers, intervention implementers and those receiving the intervention were crucial. Site leads detailed their incentive structures which included things like cash payments, certificates of training completion, reimbursement for transportation and access to training and educational resources which were given to make clinic staff feel appreciated for their commitment to the implementation.

### Implementation process

Implementation across the sites was impeded by numerous complex barriers, including patient awareness and understanding of palliative care, care coordination and communication, policy confusion and staffing challenges. The site leads discussed challenges in awareness of palliative care in general and the intervention more specifically. Most patients were unaware that they were eligible to receive palliative care or the intervention and did not know how to advocate for their preferences. Furthermore, some patients were mistrustful of the quality of primary healthcare clinics or felt their needs were out of scope, so they bypassed primary healthcare centres to seek higher levels of care. Together, this led to slow uptake of the intervention or patients refusing to participate in the research study because they felt they were not the target audience.

To pragmatically address these identified challenges, implementation strategies employed across the sites focused on what could be changed in the short term: training and mentorship for healthcare workers, strong linkages with other health services and flexibility while the intervention got off the ground. For example, in addition to intervention training, site leads discussed using WhatsApp groups for ongoing networking and mentoring to develop communities of practice. Moreover, research teams encouraged linkages with local support structures, such as specialist palliative care services in regional teaching hospitals and non-profit organisations with existing local infrastructure and networks to ease implementation. Additionally, site leads emphasised the importance of allowing for flexibility and local adaptation in the intervention protocol to account for patient, carer, provider and contextual differences. Multiple site leads described how they involved healthcare workers (ie, nurses, CHWs, physicians and other primary care clinic staff), patients, families, community groups and bespoke patient and public involvement groups to foster engagement through the intervention development, feasibility testing and beyond. Finally, site leads celebrated ongoing regional initiatives and incorporated them into the interventions rather than starting from scratch. For example, interventions incorporated items from the practical approach to care kit^19^ or worked within frameworks set out in national and local palliative care policies to facilitate implementation.

## Discussion

This synthesis of five primary palliative care intervention studies across sub-Saharan Africa demonstrates that, despite varied settings, populations and intervention designs, each study represents a feasible and acceptable model of context-specific primary palliative care. For site leads, this meant that studies met recruitment and retention targets, staff were adequately trained and retained, and interventions were able to be embedded in existing workflows seamlessly. Crucially, these findings underscore that strengthening primary palliative care across the region is not only possible but also sustainable when implementation is guided by a nuanced understanding of contextual realities and proactive strategic planning. The successful delivery of each intervention hinged on the ability of study teams to navigate diverse barriers while tailoring their approach to align with local infrastructure, workforce capacity and sociocultural dynamics.

Many successes stemmed from the intervention process domain, including incorporating feedback from key stakeholders in the development of the intervention, primary palliative care’s relative advantage over the current model of care, flexibility in the intervention protocol to allow for clinic and personal preference and low cost. Incentives and mentorship to accompany education for the healthcare workers delivering the intervention was a major facilitator to successful implementation. Mentorship schemes between primary care workers and experienced palliative care providers made interventionists more confident, provided them with support to ask questions and helped prevent burnout. This has been shown to be beneficial in palliative care delivery in other settings.^20^

Moreover, the focus on social and spiritual support in each of the interventions offered an attractive alternative to usual health services that prioritised physical concerns almost exclusively.^21^ Interventions capitalised on existing work of faith-based organisations, non-governmental organisations and local religious leaders to ensure patients and families can be easily referred as needed to ensure that their concerns are adequately addressed holistically. Rather than creating new standalone systems, implementation efforts were more effective when linked with existing regional initiatives or ongoing health system strengthening programmes. This approach not only reduced redundancy but also amplified the potential for scalability and long-term sustainability.

Though interventions were all successfully implemented, site leads highlighted major barriers to feasibility and acceptability. Importantly, the broader outer setting, including national policies, funding, prevailing health system culture that prioritises disease treatment over holistic management strongly influenced whether and how well interventions were implemented. Studies have shown that even where national policies exist, inadequate financing and fragmented funding streams limit their effectiveness, leading to inconsistent service delivery and poor integration into primary healthcare systems.^22 23^ Integrating palliative care financing into universal health coverage frameworks and developing ring-fenced budget lines within national health plans offers a potential solution to mismatched policies and budgets.^1^

A recurring and critical challenge identified across study sites was the relative ease of securing short-term research funding compared with the difficulty of sustaining service delivery once research activities conclude. While international funders were often willing to support time-bound pilot studies focused on innovation, evaluation or capacity building, this funding rarely translated into long-term financing for routine primary palliative care services. This created a structural mismatch in which evidence for effectiveness could be generated without parallel mechanisms to maintain services beyond the study period. Addressing this gap requires a deliberate shift from viewing primary palliative care as a project-based innovation to recognising it as a core health system function. Potential strategies include embedding intervention components within existing primary healthcare budgets, aligning palliative care delivery with universal health coverage benefit packages and advocating for ring-fenced domestic financing linked to national palliative care policies.^24^ Strengthening partnerships with local non-governmental and faith-based organisations, formalising referral and cofinancing arrangements and engaging ministries of health early to plan for postresearch transition have been shown to be pragmatic approaches to sustainability.^25^ Importantly, designing research studies with explicit sustainability plans, such as phased handover to health system actors, cost analyses and policy engagement milestones, may help ensure that the generation of evidence is accompanied by viable pathways for long-term service delivery rather than time-limited gains.

Even with strong national palliative care policies and sustainable funding mechanisms, national benchmarks for disease-oriented care threaten to undermine primary palliative care delivery. Primary healthcare systems and infrastructure in most sub-Saharan African countries were designed to manage and cure acute and/or infectious diseases.^26^ This has shaped the culture within these systems and socialised the societal expectations from them. As a result, policy makers, healthcare workers and patients required mental and cultural perspective shift to consider chronic serious illnesses holistically and ensure that patients’ preferences and goals drive care.^27^

Moreover, sociocultural attitudes towards death and dying, as well as entrenched gender norms around caregiving, significantly shaped both patient and provider engagement across the included studies. In many sub-Saharan African settings, death is often viewed as a taboo subject, limiting open communication about prognosis and palliative care options, and contributing to delays in seeking care or acceptance of services.^28^ These cultural perceptions can also influence healthcare providers, who may hesitate to initiate end-of-life conversations due to fears of stigma, family conflict or emotional burden.^11^ Gender roles further compound these challenges, as caregiving is predominantly assigned to women, is often unpaid and untrained and reinforces gender inequities in access to education, support and economic opportunity.^29^ This pattern echoes findings from broader literature showing that in many LMICs, the intersection of sociocultural taboos and gendered expectations creates barriers to effective palliative care delivery and uptake.^7 21^ These insights underscore the need for culturally sensitive, gender-responsive implementation strategies that address these social determinants as integral to successful palliative care scale-up.

Similarly, a key cross-cutting finding was the decisive role of sustained stakeholder engagement in facilitating successful implementation, or in other words, optimising the individual characteristics domain. Across all sites, early and ongoing buy-in from clinic staff, mid-level managers and policy makers emerged as the single most important enabler of intervention uptake and sustainability. Engagement was not limited to administrative approval but extended to fostering genuine understanding of the purpose and benefits of primary palliative care. Clinic-level buy-in was particularly effective when supported by education, mentorship and practical incentives, which together strengthened ownership of the intervention. Similarly, endorsement from mid-level managers and alignment with local health leadership priorities created momentum and embedded interventions more securely into existing workflows.

Previous research supports these findings and has shown that engagement is most effective when stakeholders, especially frontline healthcare workers and mid-level managers, are involved early in intervention codesign, have opportunities for ongoing input and receive clear evidence of benefit to patients and systems.^30 31^ Though engagement with policy makers is widely regarded as a lever for advancing palliative care, its real-world impact remains limited unless implementation is supported through sustained engagement with those who can translate national policy into actionable local strategies, like clinic leaders and healthcare workers on the ground.^32^ Building trusted relationships with community leaders, religious authorities and patient advocacy groups has also emerged as a key facilitator, helping to mitigate stigma around death and dying and reinforce local relevance and acceptability of palliative care models.^31^ These strategies align closely with the approaches adopted in our synthesis and further underscore the value of multilevel, participatory engagement in achieving sustainable change.

In terms of the inner setting, the findings from this synthesis highlight the constraints that infrastructural gaps, including lack of private spaces, inadequate technological infrastructure and workforce shortages, pose to delivery of primary palliative care. Investment in primary healthcare in many sub-Saharan African countries has been consistently poor, leaving an exhausted healthcare workforce and facilities with derelict buildings without private consultation rooms or accessible entrances.^33^ The current global drive towards universal health coverage cannot be achieved without adequate investments in primary healthcare infrastructure to ensure they are adequately designed, appropriately equipped and staffed to support patients with serious illness and their families.

### Strengths and limitations

The strength of this study lies in the use of a determinant framework to provide in-depth understanding of contextual determinants of implementation success in primary palliative care across sub-Saharan Africa. The use of qualitative methods also provided explanatory depth to the findings. Nevertheless, some factors may limit the interpretation of the findings of this study. First, as the studies included in this synthesis were purposively sampled from an existing collaboration, there is potential for selection bias. Other primary palliative care interventions have been implemented in sub-Saharan Africa outside this programme and were not included. Nevertheless, the included studies span six countries, diverse populations and varied intervention designs, allowing identification of cross-cutting implementation determinants that are likely transferable to similar low-resource primary healthcare settings. While the number of interview participants was small, this reflects the study’s reliance on expert informants and its focus on data sufficiency rather than thematic saturation. The objective was to generate sufficiently rich, triangulated implementation data to inform cross-context synthesis, rather than to exhaustively capture individual-level perspectives. Second, the use of country leads as participants meant the data collected may be more high-level and removed from operational reality of the implementation processes. However, the site leads in each country were local researchers and clinicians who worked closely with the primary healthcare workers delivering the interventions and had firsthand experience of the implementation dynamics encountered during the study. Third, the included studies evaluated unique primary palliative care interventions for different categories of patients in different countries. The heterogeneity of interventions and populations of interest made the synthesis difficult at times but also yields more generalisable findings. Finally, interviews with study site leads were not recorded to facilitate rapport building and encourage candid reflection, though this may have limited the completeness of verbatim data.

## Conclusion

In conclusion, these five study teams were able to successfully implement varied primary palliative care interventions in sub-Saharan Africa, mitigating the impact of resource limitations, political instability and a global pandemic, among other barriers. We found that success was contingent on a nuanced approach that considers key factors within intervention, individual, outer setting, inner setting and implementation process domains. Stakeholder engagement, particularly with opinion leaders and policy experts, is crucial. Furthermore, facilitation by research teams to provide support to sometimes overwhelmed healthcare workers, aligning interventions with primary healthcare values and overcoming barriers related to physical infrastructure and staff perceptions. Pragmatic strategies, such as training, mentorship, flexibility in protocols and being prepared to manage issues with patient awareness, policy confusion and staffing are important in these contexts. The multifaceted nature of primary palliative care interventions requires a nuanced and context-specific approach, and these findings can inform the understanding of challenges to anticipate within similar contexts for future studies. The findings should shape the tailoring of strategies to local realities based on nuances in each domain to overcome any challenges and foster sustainable, person-centred palliative care within primary healthcare settings.

**Table 4:** (A) Five primary palliative care interventions’ characteristics. (B) Five primary palliative care interventions’ components

Title	Target audience	Interventionist	Mode of delivery	Duration	Healthcare worker training			
(A) Five primary palliative care interventions’ characteristics								
Integrated Primary Palliative Care in Nigeria	Adults with serious illness (HIV/AIDS, TB ischaemic heart disease, cancer or stroke) and their family caregiver	Primary healthcare providers (doctors, nurses, CHWs) with at least 6 months of experience in primary care	In-person	Three months	Three days of training covering palliative care, intervention delivery and communication skills taught in lectures and role-playing exercises			
HeAlTH Systems StrEngThening (ASSET) Integrated Palliative Care and Primary Healthcare (in South Africa)	Adults with COPD or CLD experiencing symptoms	Primary healthcare providers (doctors, nurse managers)	In-person and remote	Undefined	Four weekly training sessions on PACK, communication, care planning, symptom management and psychosocial assessment			
Multimorbid Ageing Primary Palliative Care (MAPCare) (in Ghana, Malawi, Zimbabwe)	Adults 50 years and older with two or more chronic conditions, with at least one being ‘serious’ (ie, cancer, diabetes, COPD, heart failure, HIV/AIDS or stroke)	Doctors, nurses, counsellors and social workers	In-person and remote (telephone)	18 months	All healthcare workers at the clinics were trained in the principles of PC, person-centred communication, symptom assessment and management			
Person-centred care for people living with HIV in Ghana	Adults aged 20 and older with a diagnosis of HIV/AIDS for at least 6 months	Doctors, nurses, counsellors and social workers	In-person	Three months (3 monthly intervention appointments at the primary care clinic)	Three training sessions covering: (1) person-centred care delivery; (2) holistic assessment; (3) collaborative care planning; then twice-weekly clinical supervision and mentorship			
Health Systems Strengthening through Person-Centred Care (in Uganda and Zimbabwe)	Adults with highly prevalent conditions that present clinical uncertainty (ie, cancer, heart failure and COPD)	Nurses, doctors and social workers	In-person	Six months	Three days of training covering palliative care, intervention delivery and communication skills taught in lectures and simulations			
(B) Five primary palliative care interventions’ components								
Title	Physical needs assessment	Psychological needs assessment	Social and spiritual needs assessment	End-of-life care	Caregiver education	Referrals	Disease-specific training	Audio-visual material
Integrated Primary Palliative Care in Nigeria	Physical examination focused on mobility, ADLs and wounds, pain assessment	Assessment/support including counselling, relaxation therapy, person-centred goal setting and narrative therapy; referral to additional support for those with depression or anxiety	Assessment and support focusing on role, relationship and belonging needs, financial needs and practical support needs; referral to appropriate resources; spiritual concerns screening using Spirit-8 tool	Holistic end-of-life assessment and goals of care conversations	Integrated throughout the holistic assessment	Referral pathways for patients with uncontrolled symptoms, anxiety and depression and those who need additional social support	N/a	N/a
HeAlTH Systems StrEngThening (ASSET) Integrated Palliative Care and Primary healthcare (in South Africa)	Symptom assessment; patients receive care diary to record breathlessness events, emergency visits, hospitalisation and self-management techniques used	Psychological distress screening	MOS social support survey	N/a	Integrated throughout the holistic assessment; caregivers are provided with leaflets on symptoms and management	Referral to community-based services for patient and family support	Healthcare providers receive training in COPD concerns like diagnosis pathways, breathlessness, smoking cessation, respiratory exercise and inhaler techniques	Patient education leaflets on symptom management, breathlessness support services and smoking cessation; education videos
Multimorbid Ageing Primary Palliative Care (MAPCare) (in Ghana, Malawi, Zimbabwe)	Symptom assessment; patients receive care journal to track symptoms over time with thresholds to call the provider	Assessment using the IPOS tool to identify needs	Assessment using the IPOS tool to identify social and spiritual needs	Holistic end-of-life assessment and goals of care conversations	N/a	Referral pathways to tertiary care facilities and palliative care specialists	N/a	Patient self-management leaflets including journals to track symptoms
Person-centred care for people living with HIV in Ghana	Physical examination focusing on pain, symptoms, drug side effects and exhaustion	Psychosocial assessment focused on improving QoL including questions related to psychological well-being, depression and suicide, anxiety, substance abuse, grief and loss, stigma and discrimination, social isolation, employment, intimacy and sexual relationships and referral to additional resources if appropriate	Spiritual assessment using the HOPE checklist	N/a	Not described	Healthcare providers receive training in HIV-specific concerns like HIV-related stigma and discrimination, sexual health and status disclosure	Handouts for healthcare providers to use when performing a holistic assessment and developing the plan of care	
Health Systems Strengthening through Person-Centred Care (in Uganda and Zimbabwe)	Physical examination focusing on pain, symptoms, fatigue and mobility issues; tracking symptoms using IPOS	Assessment using the IPOS tool to identify needs	Assessment using the IPOS tool to identify social and spiritual needs	N/a	Nurse delivered education based on what they need to know about the prognosis, what to expect and their own selfcare	Referral pathways to tertiary care facilities and palliative care specialists	N/a	N/a

ADLs, activities of daily living; CHWs, community health workers; CLD, chronic lung disease; COPD, chronic obstructive pulmonary disease; HOPE, Hospice Outcomes and Patient Evaluation; IPOS, Integrated Palliative care Outcome Scale; MOS, Medical Outcomes Study; N/a, not applicable; PACK, Practical Approach to Care Kit; PC, palliative care; QoL, quality of life; TB, tuberculosis.

## Data Availability

Data are available upon reasonable request.
